# Adverse Effects of Methylglyoxal on Transcriptome and Metabolic Changes in Visceral Adipose Tissue in a Prediabetic Rat Model

**DOI:** 10.3390/antiox9090803

**Published:** 2020-08-31

**Authors:** Martina Hüttl, Irena Markova, Denisa Miklankova, Pavol Makovicky, Terezie Pelikanova, Ondrej Šeda, Lucie Šedová, Hana Malinska

**Affiliations:** 1 Centre for Experimental Medicine, Institute for Clinical and Experimental Medicine, 140 21 Prague, Czech Republic; mabw@ikem.cz (M.H.); irma@ikem.cz (I.M.); mild@ikem.cz (D.M.); 2 Faculty of Education, Department of Biology, J. Selye University, 94501 Komarno, Slovakia; makovicky.pavol@gmail.com; 3 Diabetes Centre, Institute for Clinical and Experimental Medicine, 140 21 Prague, Czech Republic; tepe@ikem.cz; 4 Institute of Biology and Medical Genetics, First Faculty of Medicine, Charles University and General University Hospital, 121 08 Prague, Czech Republic; ondrej.seda@lf1.cuni.cz (O.Š.); lsedo@lf1.cuni.cz (L.Š.)

**Keywords:** methylglyoxal, adipose tissue, insulin resistance

## Abstract

Excessive methylglyoxal (MG) production contributes to metabolic and vascular changes by increasing inflammatory processes, disturbing regulatory mechanisms and exacerbating tissue dysfunction. MG accumulation in adipocytes leads to structural and functional changes. We used transcriptome analysis to investigate the effect of MG on metabolic changes in the visceral adipose tissue of hereditary hypetriglyceridaemic rats, a non-obese model of metabolic syndrome. Compared to controls, 4-week intragastric MG administration impaired glucose tolerance (*p* < 0.05) and increased glycaemia (*p* < 0.01) and serum levels of MCP-1 and TNFα (*p* < 0.05), but had no effect on serum adiponectin or leptin. Adipose tissue insulin sensitivity and lipolysis were impaired (*p* < 0.05) in MG-treated rats. In addition, MG reduced the expression of transcription factor *Nrf2* (*p* < 0.01), which controls antioxidant and lipogenic genes. Increased expression of *Mcp-1* and *TNFα* (*p* < 0.05) together with activation of the SAPK/JNK signaling pathway can promote chronic inflammation in adipose tissue. Transcriptome network analysis revealed the over-representation of genes involved in insulin signaling (*Irs1, Igf2, Ide*), lipid metabolism (*Nr1d1, Lpin1, Lrpap1*) and angiogenesis (*Dusp10, Tp53inp1*).

## 1. Introduction

Metabolic changes and dysregulation in white adipose tissue (WAT) can trigger and considerably contribute to the development of metabolic diseases, such as metabolic syndrome, type 2 diabetes and associated complications. Alterations in WAT occur in the early stages of these disorders and can even precede weight gain. Accordingly, WAT is considered an important therapeutic target. As a result of various types of adipokine secretion, WAT regulates lipid and glucose metabolism, food intake and energy expenditure [1,2]. It is also one of the first organs in which insulin resistance develops as a consequence of excessive and impaired lipid accumulation, leading to hypoxia and activation of inflammatory pathways. 

Apart from hypoxia, glycation is among the early pathological factors of adipose tissue dysfunction [3]. It has been shown that glycation processes can induce structural and morphological alterations in adipose tissue [4], and also impair vascular microcirculation (blood flow), leading to hypoxia and insulin resistance [5].

Dicarbonyl stress plays a role in the pathological mechanism of glycation by causing excessive generation of toxic dicarbonyl metabolites such as methylglyoxal (MG) and impairing detoxification of the glyoxalase enzymatic system, particularly glyoxalase-1 (Glo-1) [6]. Excessive dicarbonyl generation leads to AGE production, activates inflammatory processes, increases oxidative stress and impairs glucose tolerance, all of which cause metabolic and vascular changes in different tissues. MG is recognized as a trigger for the development and progression of diabetic complications [7]. It is also a particularly important factor in the development of microvascular complications [8] and can impair insulin signaling and secretion [9].

The glyoxalase system member Glo-1 provides the primary defense against MG glycation. It has been reported that *Glo-1* overexpression prevents vascular ageing and ameliorates angiogenic defects in endothelial cells [10]. Although the exact role of Glo-1 in adipose tissue metabolism is unclear, recent studies report that decreased *Glo-1* expression and activity through hypoxia and inflammatory signaling in adipose tissue drives dicarbonyl stress in obese patients [11]. 

MG accumulation in adipocytes after exposure to MG causes structural and functional changes in adipose tissue [4] independently of obesity; these alterations may precede the onset of metabolic syndrome and type 2 diabetes (T2D). Indirectly, MG can disturb and impair different signaling pathways and also trigger epigenetic changes [12]. However, the exact mechanism underlying the pathophysiological role of MG and glyoxalase-1 in adipose tissue is not yet fully understood.

In this study, we used transcriptome profiling to investigate the effect of MG on metabolic dysfunction in visceral adipose tissue using a prediabetic model, the hereditary hypetriglyceridaemic (HHTg) rat. Originating from the Wistar rat, this strain exhibits dyslipidaemia, insulin resistance, ectopic lipid deposition and low-grade chronic inflammation in the absence of obesity and hyperglycaemia [13]. 

Adipose tissue dysfunction can begin during the early stages of T2D development and before the onset of hyperglycaemia. It has been reported that both hyperglycaemia and dyslipidaemia lead to excessive MG production. As we previously reported, dyslipidaemia in a prediabetic rat model led to elevated levels of MG and MG-derived advanced glycation end products (AGEs) in tissues [14].

## 2. Materials and Methods 

### 2.1. Animals and Diet

All experiments were performed in agreement with the Animal Protection Law of the Czech Republic (311/1997), which complies with European Community Council recommendations (86/609/EC) for the use of laboratory animals, and approved by the Ethics Committee of the Institute for Clinical and Experimental Medicine, Prague.

All experimental procedures were carried out using 5-month-old male hereditary hypertriglyceridaemic (HHTg) rats (8 animals in each group) supplied by our institute. The rats were fed a standard laboratory diet (23% protein, 43% starch, 7% fat, 5% fiber and a 1% vitamin and mineral mixture; Bonagro, Czech Republic) and kept under temperature- and humidity-controlled conditions based on a 12 h/12 h light–dark cycle. The animals had free access to food and drink at all times. Before starting the trial, there were no differences in initial body weight (398.2 ± 15.6 versus 393.9 ± 9.8 g), glucose (6.5 ± 0.6 versus 6.9 ± 0.5 mmol/l), or serum triglycerides (7.688 ± 0.849 versus 7.608 ± 0.594 mmol/l) between experimental groups. In the MG-treated group of HHTg rats, MG (Sigma-Aldrich, catalog number M0252) was administered intragastrically three times a week at a dose of 0.5 mg/kg bodyweight for four weeks. In the control group, water was administered intragastrically over the same period. At the end of the study, animals were sacrificed by decapitation in a postprandial state. Aliquots of serum and tissue samples were rapidly removed, weighted, frozen in liquid nitrogen and stored at −80°C for analysis.

### 2.2. Biochemical Analysis

Serum levels of glucose, triglycerides and non-esterified fatty acids (NEFA) were measured using commercially available kits (Erba Lachema, Czech Republic; Roche Diagnostics, Germany). Serum insulin, HMW adiponectin, MCP-1, TNFα and leptin concentrations were determined using rat ELISA kits (Mercodia AB, Sweden; MyBioSource, USA; eBioscience, USA; BioVendor, CZ). Concentrations of MG in serum and adipose tissue were determined after derivatization with 1,2-diaminobenzene using the HPLC method and fluorescence detection as previously described [15]. 

### 2.3. Basal and Insulin-Stimulated Glucose Utilisation in Adipose Tissue and Lipolysis

For measurement of insulin-stimulated incorporation of glucose into lipids, epididymal adipose tissue were incubated for 2 h in 95% O_2_ + 5% CO_2_ in Krebs-Ringer bicarbonate buffer (pH 7.4) containing 0.1 μCi/mL of ^14^C-U glucose, 5 mmol/L of unlabelled glucose and 2.5 mg/mL of bovine serum albumin (Fraction V, Sigma, Czech Republic) with and without 250 μU/mL of insulin. Lipids was extracted followed by determination of insulin-stimulated incorporation of glucose into lipids. In epididymal adipose tissue, adrenaline-stimulated lipolysis was measured ex vivo according to the release of NEFA into the incubating medium. 

### 2.4. Fatty Acid Composition

Extraction, separation, and methylation of visceral adipose tissue phospholipids were performed as previously described [16]. Briefly, total lipids were extracted with dichloromethane methanol using a modification of the Folch method. Phospholipids were isolated by thin-layer chromatography using hexane-diethyl ether-acetic acid (80:20:3, *v*/*v*) as the solvent system. Fatty acids (FAs) in phospholipids were converted to methyl esters using a 1% solution of Na in methanol. Methyl esters were eluted with hexane and separated by gas chromatography using the Hewlett Packard GC system, hydrogen as the carrying gas, a flame ionization detector, and a Carbowax fused silica capillary column (Varian, Palo Alto, CA, USA) [17]. Individual peaks of FA methyl esters were identified by comparing retention times with authentic standards (mix of standard FAs, Restek Corporation, PA, USA). FA proportions (spectrum of the 18 main FAs of interest) are given as the relative percentage of the sum of FAs analyzed. 

### 2.5. Transcriptome and Gene Expression Profiling

Transcriptome assessment of white adipose tissue was performed using the GeneChip^®^ Rat Gene 2.1 ST Array Strip on the Affymetrix Gene Atlas System (Thermo Fisher Scientific, Waltham, MA, USA). Total RNA was extracted from an epididymal fat pad using phenol-chloroform and purified using the RNeasy Mini Kit (Qiagen, Valencia, CA, USA). The quality and integrity of total RNA were evaluated on the Agilent 2100 Bioanalyzer system (Agilent, Palo Alto, CA, USA). Only samples surpassing the minimum quality threshold (RIN > 8.0) were used for subsequent transcriptome assessment. The whole procedure, comprising several phases of reverse transcription, was performed according to manufacturer protocol. Microarray data were deposited in the ArrayExpress database (www.ebi.ac.uk/arrayexpress) under accession number E-MTAB-9013.

Microarray results were validated by qPCR. Reverse transcription and quantitative real-time PCR analysis was performed using the TaqMan RNA-to-C_T_ 1-Step Kit, the TaqMan Gene Expression Assay (Applied Biosystems, Foster City, CA, USA) and the ViiA^TM^ 7 Real-Time PCR System (Applied Biosystems, USA). Relative expression was determined after normalization against β-actin as an internal reference and calculated using the 2^−^^ΔΔCt^ method.

### 2.6. Histological Methods

After sampling, adipose tissue samples were fixed immediately in 4% formaldehyde solution for 48 h and processed in paraffin blocks using standard techniques. Three-to-five-μm-thick slices were cut from each sample using a microtome. The first slices were stained with haematoxylin-eosin (DiaPath, Martinengo, Italy). To detect glycoconjugates and PAS-positive material, the second sections were stained using the PAS-Hotchkiss-McManus methodology (DiaPath, Martinengo, Italy). The prepared slides were then evaluated by a veterinary histopathologist. 

### 2.7. Statistical and Pathway Analysis

All data were statistically evaluated using the unpaired Student’s *t*-test, with categorical variables analyzed using Fisher’s exact test (used statistical software Graph-Pad InStat 3.1). Before beginning the study, χ^2^ test was used to examine qualitative variables. Statistical significance was defined as *p* ˂ 0.05, with data expressed as the mean ± SD.

For transcriptome data, hybridization and quality control were evaluated using the Affymetrix Expression Console (Thermo Fisher Scientific, Waltham, MA, USA). Data were then normalized (robust multi-array average (RMA)). Gene expressions were compared between the MG-treated group and the control group using analysis of variance with multiple comparison adjustment and the false discovery rate method (FDR < 0.05) as implemented in PARTEK Genomics Suite 7 (Partek Inc., St. Louis, MI, USA). Transcripts significantly differentially expressed by more than 1.2-fold between both groups (FDR ˂ 0.1) were processed for gene enrichment and network/pathway analysis using Ingenuity Pathway Analysis software (Qiagen Redwood City, Inc., Redwood City, CA, USA).

## 3. Results

At the beginning of the study and before MG administration, we observed no differences in body weight, serum glucose or triglycerides between both groups of HHTg rats. MG administration did not affect food or drink consumption. 

In HHTg rats, MG administration did not alter body weight. However, it did aggravate glucose intolerance, with non-fasting glucose and serum insulin significantly increased in MG-treated animals (Table 1). 

### 3.1. Effects of Methylglyoxal on Insulin Sensitivity and Lipolysis

**Figure 1:**
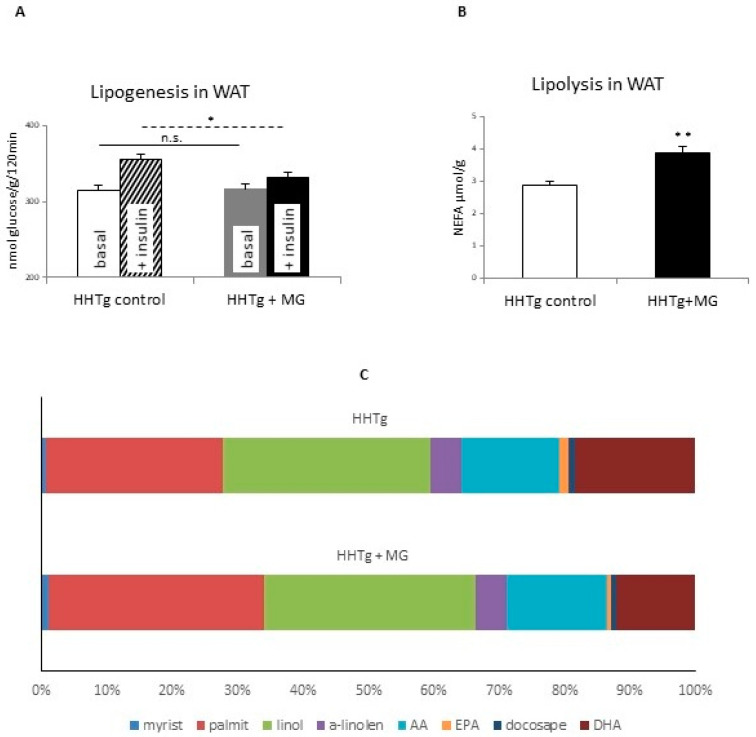
Basal and insulin-stimulated ^14^C-U glucose incorporation into lipids (**A**), adrenaline stimulated lipolysis (**B**) and phospholipid fatty acid composition in visceral adipose tissue (**C**) in HHTg (hereditary hypetriglyceridaemic) rats treated with methylglyoxal compared to control HHTg rats. Data are expressed as means (SD) and analyzed using the two-tailed unpaired Student’s *t*-test. * denotes *p* < 0.05, ** denotes *p* < 0.01, n.s. denotes non-significant.

Although MG administration did not alter adiposity, insulin sensitivity in adipose tissue due to insulin stimulation was markedly impaired (Figure 1A). However, serum adiponectin levels (Table 1) and skeletal muscle insulin sensitivity (data not shown) in MG-treated rats were not different to controls. While MG administration increased adrenaline-stimulated lipolysis (Figure 1B), NEFA concentrations only increased slightly [18]. 

### 3.2. Effects of Methylglyoxal on Fatty Acid Composition of Phospholipids in Visceral Adipose Tissue

MG-treated rats exhibited a substantial shift in fatty acid composition in visceral adipose tissue phospholipids (Figure 1 and Table 2) compared to the control group. While the proportion of saturated fatty acids, especially palmitic (16:00) and myristic (14:00) acid, significantly increased, we observed a decrease in proportions of the n-3 PUFAs α-linolenic acid (18:3n3), eicosapentaenoic acid (EPA) (20:5n3) and docosahexaenoic acid (DHA) (22:6n3). These findings point to a possible negative influence on membrane fluidity and insulin signaling. There was also an increase in proportions of the n-6 PUFAs, linoleic (18:2n6) and arachidonic (20:4n6) acids, which can contribute to oxidative stress and inflammation.

### 3.3. Effects of meThylglyoxal on Dicarbonyl Stress and Inflammatory and Hypoxic Parameters

**Figure 2:**
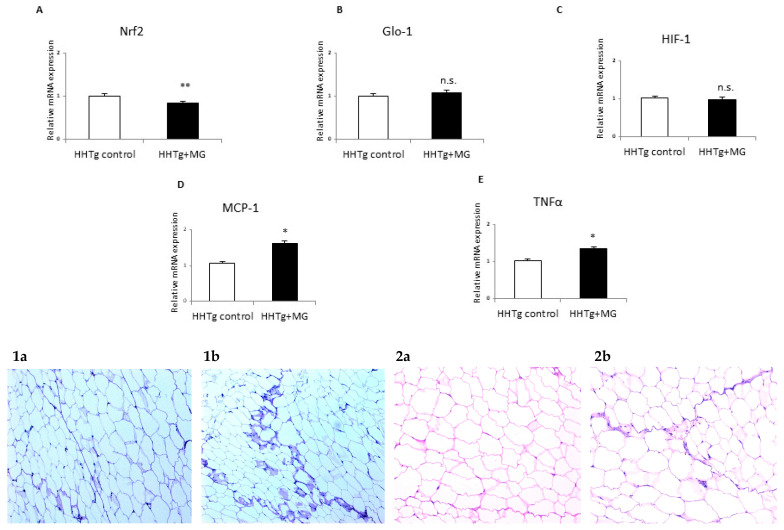
Effects of methylglyoxal (MG) treatment on dicarbonyl stress, inflammation and hypoxia parameters in visceral adipose tissue. Relative mRNA expression of *Nrf2* (Panel **A**), *Glo-1* (Panel **B**), *Hif-1* (Panel **C**), *MCP-1* (Panel **D**) and *TNFα* (Panel **E**) in visceral adipose tissue of MG-treated rats compared to HHTg (hereditary hypetriglyceridaemic) control rats; Nrf2—nuclear factor erythroid 2-related factor 2; Glo-1—glyoxalase 1; HIF-1—hypoxia-inducible factor 1; MCP-1—monocyte chemoattractant protein 1; TNFα—tumor necrosis factor α. Data are expressed as means (SD) and analyzed using the two-tailed unpaired Student’s *t*-test. * denotes *p* < 0.05, ** denotes *p* < 0.01, n.s. denotes non-significant. View of visceral adipose tissue PAS-positive glycoconjugates (**1a**), (**1b**), (**2a**), (**2b**).

After four weeks of MG administration, concentrations of circulating MG in serum and MG in visceral adipose tissue had elevated significantly (+52% and +37%, respectively) (Table 1). In adipose tissue, there were no differences in relative mRNA expression and activity of *Glo-1*, an enzyme involved in MG degradation. However, MG administration significantly reduced relative mRNA expression of transcription factor *Nrf2* (Figure 2), which controls antioxidant and lipogenic genes as well as *Glo-1* gene expression.

Compared to controls, MG administration markedly increased serum levels of pro-inflammatory factors MCP-1 and TNFα, but serum levels of leptin and IL-6 were not affected (Table 1). Relative mRNA expression of *Mcp-1* and *TNFα* in adipose tissue was elevated in MG-treated rats compared to controls, but there were no changes in *Hif1* gene expression after MG administration (Figure 2). Inflammatory infiltrates and the presence of PAS-positive glycoconjugates in visceral adipose tissue of MG-treated animals were verified based on histological investigation (Figure 2).

### 3.4. Effects of Methylglyoxal on the Visceral Adipose Tissue Transcriptome

Based on comparative transcriptome analysis of visceral adipose tissue, we identified 78 transcripts, including 66 annotated genes coding for protein products or functional RNAs, as differentially expressed (FDR < 0.05) in MG-treated HHTg rats, with 51 relatively upregulated and 27 downregulated transcripts. As shown in Supplementary Table S1, among the top upregulated genes in MG-treated rats were nuclear receptor subfamily 1, group D, member 1 (*Nr1d1*, 2.17-fold, *p* = 6.06E-07) and regulator of G-protein signaling 2 (*Rgs2*, 1.66-fold, *p* = 1.02E-05). The top genes downregulated by MG included cytokine-inducible SH2-containing protein (*Cish*, −5.3-fold, *p* = 2.15E-06) and insulin receptor substrate 1 (*Irs1*, −1.65-fold, *p* = 2.18E-05).

To identify upstream regulators that might potentially affect MG-induced transcriptome alterations, we used an algorithm based on the expected causal effects between upstream regulators and their targets using Ingenuity Pathway Analysis software [19]. The following most likely upstream regulators were identified: APP—amyloid beta precursor protein (*p* = 4.12E-06), ZBTB20—zinc finger and BTB domain-containing 20 (*p* = 9.66E-06), PTGER4—prostaglandin E receptor 4 (*p* = 4.30E-05) and PNPLA2—patatin-like phospholipase domain-containing 2 (*p* = 3.07E-04). Analysis of toxicity lists and functions revealed differences in gene expression associated with p53 signalling (*p* = 3.39E-03) and NFkB signaling (*p* = 3.62E-02) or the involvement of white adipose tissue morphology (*p* = 1.70E-04).

**Figure 3:**
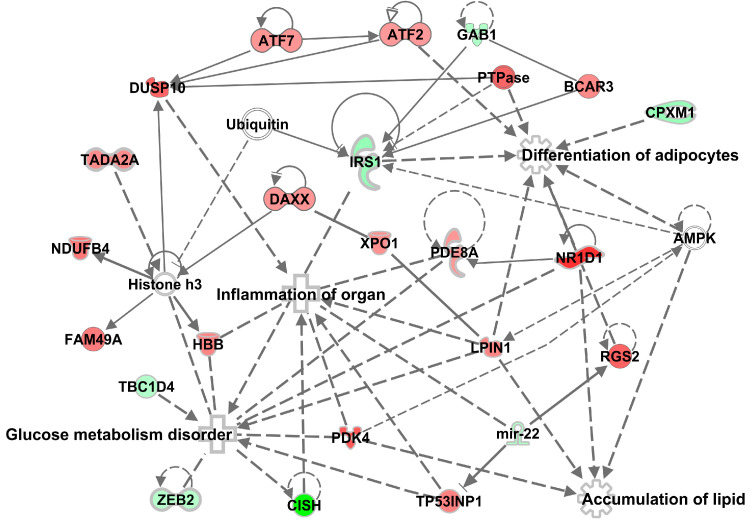
Mechanistic network showing the highest score for a set of significantly differentially expressed genes in MG (methylglyoxal)-treated rats. The level of change in expression is highlighted in shades of green (MG downregulation) and red (MG upregulation). Network derivation was performed using Ingenuity Pathway Analysis software.

**Figure 4:**
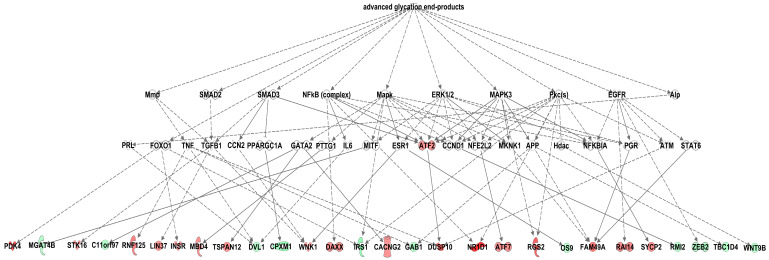
Highest-scoring Causal Network connecting multiple levels of upstream regulators to the significantly differentially expressed genes in white adipose tissue of MG-treated HHTg (hereditary hypetriglyceridaemic) male rats compared to controls. The effect of MG (methylglyoxal) on expression is shown in shades of green (downregulation) or red (upregulation). Full and dashed lines indicate known direct and indirect interactions between the upstream regulator and its downstream target. Derivation of the network was performed using Ingenuity Pathways Analysis.

**Figure 5:**
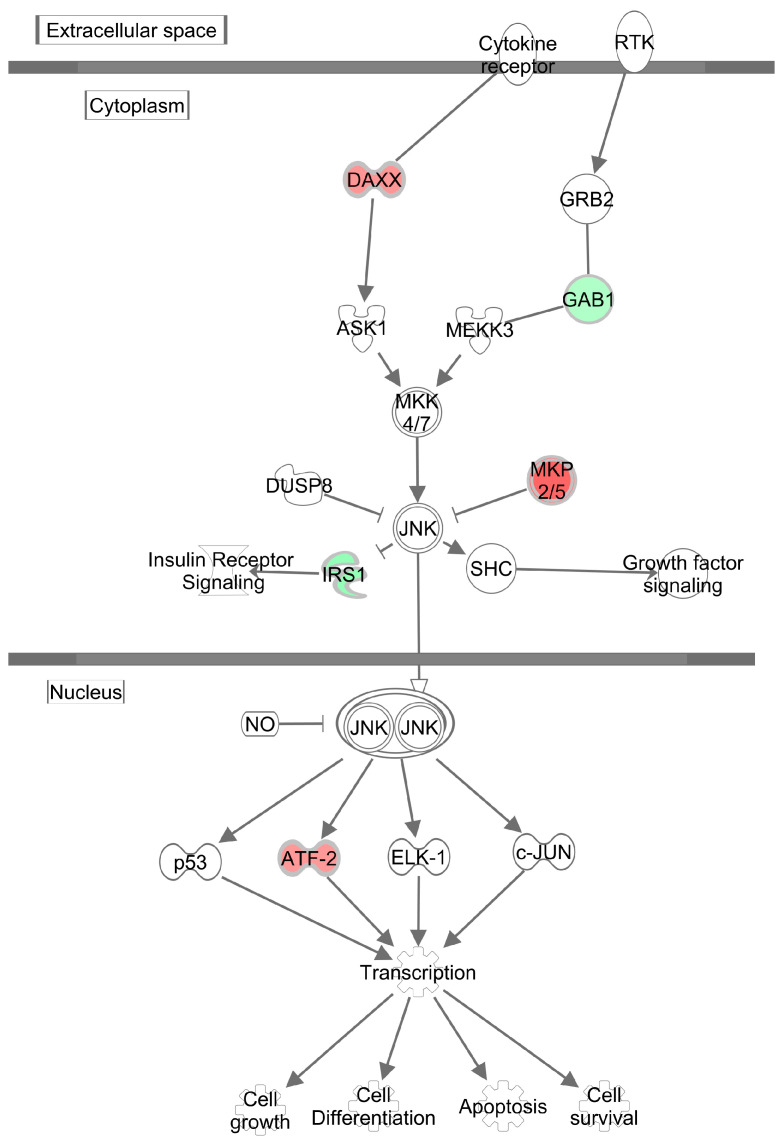
Simplified scheme of Sapk/Jnk signaling pathway showing significant enrichment (Benjamini–Hochberg corrected *p* = 1.11E-03) by transcripts differentially expressed in in white adipose tissue of MG (methylglyoxal)-treated HHTg (hereditary hypetriglyceridaemic) male rats compared to controls. The effect of MG on expression is shown in shades of green (downregulation) or red (upregulation). Created with Path Designer module, Ingenuity Pathways Analysis.

Network analysis revealed over-representation of genes involved in pathways related to glucose metabolism disorder (*Irs1*, *Cish*, *Pdk4*, *Rgs2*, *Xpo1*), inflammation (*Cish*, *Nr1d1*, *Dusp10*, *Pde8a*, *Tp53inp1*, *Pdk4*, *ubiquitin*), accumulation of lipid (*Nr1d1*, *Lpin1*, *Pdk4*) and adipocyte differentiation (*Nr1d1*, *Lpin1*, *Rgs2*, *Irs1*, *Cpxm1*), all of which are implicated in MG-induced transcriptome alterations (Figure 3). The most significant “Causal” network (small hierarchical network of regulators that control the expression of the dataset targets; *p* = 9.48E-06, network bias-corrected *p* value = 8.0E-04) featured increase in advanced glycation end-products as a major predicted master regulator based on 29 transcripts differentially expressed in MG-treated rats (Figure 4). The canonical pathway analysis revealed activation (on the level of mRNA) of a single pathway, the SAPK/JNK signaling pathway (*p* = 1.15E-05), which is involved in proliferation, apoptosis, metabolism and DNA repair (Figure 5). The above transcriptome data indicate the effects of MG on visceral adipose tissue, primarily on various cell signaling and regulatory pathways containing G-proteins, MAPK, ERK, PKC, SREBPs and NFκB.

## 4. Discussion

In this study, MG-induced glycation processes significantly affected visceral adipose tissue functions at both metabolic and transcriptome levels in prediabetic rats. MG administration induced dicarbonyl stress comparable to levels in poorly controlled diabetes, correlating with the development of severe diabetic complications. MG-treated animals exhibited markedly increased dicarbonyl stress parameters in adipose tissue. In addition to increased MG levels, decreased gene expression of transcription factor *Nrf2* in adipose tissue can contribute to increased dicarbonyl stress. Nrf2 is a master regulator of antioxidant response element (ARE)-dependent genes, which include many antioxidant and detoxification enzymes [20]. It also regulates oxidative stress responses and participates in MG detoxification by controlling gene expression of the MG detoxification enzyme Glo-1. However, Nrf2 transcription factor also regulates networks of genes controlling diverse processes and can play a critical role in lipid and glucose metabolism. Recent data indicate that Nrf2 may regulate the formation and function of WAT via lipid metabolism of adipocytes, adipogenesis, lipogenesis, lipolysis and insulin signalling [21]. The mechanism by which MG can reduce *Nrf2* gene expression is not exactly known, however increased oxidative stress, endoplasmic reticulum stress or direct glycated impairment may be involved.

In our study, MG-induced glycated processes contributed to metabolic dysfunction in adipose tissue, leading to increased lipolysis and decreased lipogenesis. Transcription factor Nrf2 is understood to regulate both processes, which are significant drivers of adipocyte lipid metabolism. Nrf2 regulates lipolysis by enhancing the phosphorylation of lipolytic enzymes. Lipolysis is exceptionally sensitive to insulin action, and diminishing the antilipolytic effect of insulin on adipose tissue elevates the release of NEFA into the bloodstream [22]. However, in contrast to previous findings [5], levels of NEFA only slightly increased in our study.

Although MG administration had no effect on circulating triglyceride or ectopic triglyceride accumulation [18], transcriptome analysis did reveal the involvement of MG in the regulation of adipose tissue lipid metabolism. Upregulated *Lpin1* positively affects triglyceride biosynthesis control in the endoplasmic reticulum, while *Nr1d1* regulates lipid metabolism by influencing SREBPs and PPARα. On the other hand, downregulated *Lrpap1*, which encodes a protein that interacts with LDL receptors, is involved in the alteration of circulating lipids, as observed after MG exposure in our study [18] and in others [4,5].

Based on transcriptome profiling, decreased insulin sensitivity in adipose tissue after MG exposure (measured by lipogenesis) was accompanied by changes to genes involved in the insulin signalling cascade, particularly *Irs1*. MG administration markedly changed genes associated with insulin signalling (*Irs1*, *Rgs2*), action (*Igf2*) and glucose vs. fatty acid utilization (*Pdk4*).

Nrf2 is understood to participate in the regulation of IRS-1 phosphorylation, but the exact role of Nrf2 in insulin signaling remains inconclusive. While Nrf2 protects cells against oxidative damage and can improve glucose homeostasis and insulin resistance, it can also impair insulin-stimulating ROS signaling [23]. Thus, the aggravation of insulin resistance in MG-treated rats may be due to interference with insulin receptor signaling.

Weakened insulin action in adipose tissue after MG exposure can lead to negative alterations in FA profiles in adipose tissue membrane phospholipids. An increased fraction of saturated fatty acids together with reduced n-3 PUFA markedly affect membrane fluidity, contributing to the impairment of insulin signaling. The relationship between fatty acid profiles and insulin action has been observed in clinical studies of diabetic patients [24] and elderly obese men [25]. Changes in FA composition, particularly linoleic, arachidonic and DHA fatty acids, play a role in modulating insulin action in peripheral tissues. A positive association between insulin resistance and enriched palmitic acid and depleted essential n-3 PUFA in adipose tissue has also been found. In addition, alterations in fatty acid profiles in visceral adipose tissue membrane phospholipids are related to intracellular metabolism and macrophage polarization [26]. In our previous human study, profiles of palmitic and palmitoleic acid in WAT correlated positively with the proportion of pro-inflammatory macrophages and negatively with the n-3 PUFA profile, correlations connected with chronic inflammation in adipose tissue.

Based on transcriptome profiling, our results reveal that glycation in adipose tissue after MG exposure affected gene expression associated with adipogenesis regulation, leading to upregulation of *Atf2 and Tp53inp1* and downregulation of *Cpxm1*. Via these mechanisms, glycation can contribute to the impairment of adipocyte differentiation and proliferation.

Furthermore, Nrf2 may function as a positive regulator in angiogenesis [21]. Accordingly, markedly decreased gene expression of Nrf2 in adipose tissue in MG-treated rats can impair adipocyte differentiation. Suppression of Nrf2 activity attenuates adipogenesis by reducing PPRAγ in 3T3 cells [27]. Moreover, adipocyte differentiation and function can be affected by cellular redox status, which may be influenced by Nrf2.

In our study, adipose tissue metabolic dysfunction after MG administration activated cellular inflammatory and stress-response pathways, particularly SAPK/JNK signaling. Activation of these signaling pathways increases gene expression and secretion of the pro-inflammatory cytokines MCP-1 and TNFα, leading to chronic inflammation in adipose tissue and decreased glucose and lipid uptake in adipocytes. MCP-1 is a key chemokine responsible for monocyte recruitment and accumulation in adipose tissue, mechanisms that lead to the secretion of cytokines, particularly TNFα [28]. The secreted cytokines TNFα and IL-6 cause insulin resistance in adipocytes through IKKβ and JNK1 kinase pathway activation, which interfere with insulin signaling via phosphorylation and subsequent IRS1 inactivation [29]. TNFα can activate JNKs and inhibit IRS-1 phosphorylation, leading to insulin resistance [30]. TNFα activates MAPK and JNK signaling in adipocytes associated with lipolysis regulation [31]. In a study of non-obese diabetic rats, MG accumulation in adipose tissue resulted in increased expression of MCP-1 and caused apoptotic and angiogenic alterations in adipose tissue [4].

Our transcriptome profiling revealed downregulation of *Cish*, which is involved in the negative regulation of cytokine signaling through the JAK-STATS pathway. Upregulation of intracellular receptor *Nr1d1* in MG-treated rats activates signaling pathways through the MAPK superfamily, particularly MAPK4, thus negatively affecting the regulation of inflammatory pathways.

In studies involving cell cultures, MG and MG-derived AGEs have been shown to promote VEGF and MCP-1 expression through the activation of p38 MAPK signaling [32,33]. AGE can promote inflammation via RAGE, TLR4 and other receptors that regulate NFκB activity [34]. Despite correlations found previously between TNFα and total body fatness [35] as well as circulating MCP-1 and BMI [36], in our study, increased pro-inflammatory markers and activated pro-inflammatory pathways after MG administration were not associated with any changes in adiposity. Interestingly, there were no changes in adiposity in MG-treated rats.

The activation of inflammatory signaling pathways after MG treatment can also contribute significantly to alterations in fatty acid composition in adipose tissue phospholipids, elevating the profiles of saturated (palmitic and myristic) and pro-inflammatory PUFAs (arachidonic) and markedly decreasing the profiles of anti-inflammatory PUFAs (EPA and DHA). Saturated FAs activate TLR4 receptors and potentiate inflammation and insulin resistance in adipocytes via the TLR4/PI3K/PKB signaling pathway [2,37].

Inflammatory processes and hypoxia in adipose tissue can also contribute to the dysregulation of microRNAs [38]. Our transcriptome profiling results indicate the downregulation of miR-22 in adipose tissue after MG exposure. Dysregulation of miR-22 can upregulate inflammatory cytokines, promote apoptosis and, in particular, regulate hypoxia signaling. In another study, downregulation of miRNA-22 was associated with upregulation in expression of the pro-inflammatory cytokines TNFα, IL-6, IL-1b and IL-18 through the NFκB and MAPK pathways [39]. Glycation and other metabolic disorders in adipose tissue after MG exposure can lead to hypoxia—a condition that promotes insulin resistance, decreases glucose and lipid uptake in adipocytes, and activates chronic inflammation. In our study, we found no differences in *Hif-1* expression in adipose tissue of MG-treated rats, possibly indicating that hypoxia may be the consequence of other metabolic changes in adipose tissue. For instance, dysregulation of miR-22 may be one of the initial changes that potentiate hypoxia in adipocytes after MG exposure.

According to recent findings, MG is considered a trigger for the development and progression of microvascular diabetic complications preceding AGE formation and the onset of hyperglycaemia. In a study of non-obese diabetic rats with hyperglycaemia, glycated processes in adipose tissue after MG exposure not only impaired microcirculation in epididymal adipose tissue but also led to hypoxia and insulin resistance [5]. Based on our transcriptome profiling results, downregulation of the *RGS2* gene, which is involved in the regulation of angiotensin-activated signaling [40], indirectly contributed to vascular complications in adipose tissue after MG exposure. Direct glycation processes altered and impaired the structure and function of intracellular and extracellular matrix proteins and led to microvascular changes in adipose tissue. MG-modified proteins are understood to be less efficient at cell attachment and linked to decreased vessel functionality [3]. In our study, we confirmed the presence of PAS-positive glycoconjugates in adipose tissue in MG-treated rats. Although these direct glycation processes probably require higher MG levels than those that activate signaling pathways, they nevertheless play a key role in the development of microvascular complications in adipose tissue. Moreover, insulin glycation impairs the ability to bind to, and activate, receptors, thus contributing to reduced insulin action in peripheral tissues.

Our study is the first to demonstrate the metabolic effect of MG on the adipose tissue transcriptome. Acknowledged as a key factor in adipose tissue dysfunction, glycation can occur as early as in the initial stages of T2D development and even before the onset of hyperglycaemia. MG-induced glycation causes metabolic, vascular and structural changes in adipose tissue independently of hyperglycaemia and visceral fat mass. Changes in adipose tissue, particularly insulin resistance, after MG-induced glycation not only contribute to the development of disorders in other tissues but also potentiate whole-body insulin resistance. Our model confirmed these MG-induced endogenous changes independently of any impacts due to a high-calorie diet.

According to our results, *Nr1d1* was the most significantly affected gene in MG-treated rats. This nuclear receptor encodes a transcription factor involved in the regulation of genes that function in metabolic, inflammatory and vascular processes, including lipid metabolism, adipogenesis, gluconeogenesis and the inflammatory response.

## 5. Conclusions

Our results demonstrate that MG-induced glycation contributes to metabolic, vascular and structural changes in adipose tissue independently of hyperglycaemia and visceral fat mass. In our rat model of metabolic syndrome, MG exposure impaired adipose tissue insulin sensitivity and potentiated inflammation at both transcriptome and metabolic levels, pointing to the possible role of MG in adipose tissue dysfunction. This mechanism operates directly by glycation as well as indirectly by signaling and regulating the pathways that promote insulin resistance, vascular dysfunction and inflammation, especially involving Nr1d1, Irs1 genes, Nrf2 transcription factor and SAPK/JNK signaling pathway.

Although direct MG-induced glycation has a greater effect on the development of microvascular complications in adipose tissue, inflammation and insulin resistance also play a role in MG activation of signaling pathways.

## Figures and Tables

**Table 1:** Metabolic and inflammatory parameters in serum and tissue in hereditary hypetriglyceridaemic (HHTg) rats and in rats supplemented with methylglyoxal (HHTg + MG).

	HHTg	HHTg + MG	*p* ˂
body weight (g)	402 ± 22	381 ± 8	n.s.
adiposity (g/100g bwt)	1.85 ± 0.29	1.87 ± 0.35	n.s.
non-fasting glucose (mmol/L)	7.7 ± 0.3	8.6 ± 0.24	0.01
insulin (nmol/L)	0.24 ± 0.08	0.42 ± 0.05	0.05
serum triglycerides (mmol/l)	4.34 ± 0.74	3.21 ± 1.24	n.s.
methylglyoxal in serum (nmol/mL)	0.25 ± 0.03	0.37 ± 0.03	0.01
methylglyoxal in adipose tissue (nmol/mg)	0.63 ± 0.09	0.86 ± 0.13	0.05
HMW adiponectin (μg/mL)	2.90 ± 0.96	2.78 ± 1.14	n.s.
leptin (ng/mL)	2.15 ± 1.07	2.13 ± 1.42	n.s.
IL-6 (pg/mL)	87.2 ± 21.78	99.9 ± 11.78	n.s.
MCP-1 (ng/mL)	85.25 ± 40.20	124.88 ± 29.65	0.05
TNFα (pg/mL)	0.78 ± 0.04	2.11 ± 0.33	0.05

Data are given as the mean ± SD, with n = 8 for each group; Statistical significance was calculated using the two-tailed unpaired Student’s *t*-test; HMW adiponectin = High Molecular Weight adiponectin, IL-6 = interleukin-6, MCP-1 = monocyte chemoattractant protein-1, TNFα = tumor necrosis factor α.

**Table 2:** Effects of methylglyoxal on the proportion of phospholipid fatty acids in visceral adipose tissue in HHTg (hereditary hypetriglyceridaemic) rats and in methylglyoxal-treated HHTg rats (HHTg + MG).

	HHTg	HHTg + MG	*p* ˂
	% of total fatty acids	% of total fatty acids	
14:00	0.40 ± 0.18	0.76 ± 0.29	0.02
16:00	16.68 ± 5.14	24.05 ± 4.01	0.02
18:2n6	19.60 ± 1.85	23.17 ± 1.51	0.01
20:4n6	9.36 ± 2.39	10.76 ± 2.08	n.s.
18:3n3	3.24 ± 0.96	2.12 ± 0.40	0.05
20:5n3	0.91 ± 0.21	0.44 ± 0.14	0.01
22:6n3	13.15 ± 2.57	5.57 ± 1.35	0.001
SFA	33.35 ± 3.82	39.92 ± 2.96	0.01
PUFA n6	31.44 ± 3.89	35.59 ± 3.08	n.s.
PUFA n3	18.42 ± 2.37	8.80 ± 1.50	0.001
Anti-inflammatory index	171.20 ± 32.54	65.87 ± 14.46	0.001

Data are given as the mean ± SD, with n = 8 for each group. The relative concentration of each group was calculated as the proportion of all fatty acids detected to the percentage of total fatty acids. Statistical significance was calculated using the two-tailed unpaired Student’s *t*-test. Anti-inflammatory index = (22:6 n3 + 22:5 n3 + 20:3 n6 + 20:5 n3)/20:4 n6.

## Supplementary Materials

The following are available online at https://www.mdpi.com/2076-3921/9/9/803/s1, Table S1: List of transcripts significantly differentially expressed (FDR < 0.05) in white adipose tissue of adult HHTg male rats treated with methylglyoxal vs. control. ID refers to Affymetrix transcript ID, fold change indicates the change of expression in MG-treated vs. control rats. The analysis was performed using Partek Genomics Suite 7 (Partek Inc., St. Louis, MI, USA).

## Abbreviations

AGEs:	advanced glycation end products;
DHA:	docosahexaenoic acid;
EPA:	eicosapentaenoic acid;
ERK:	extracellular signal-regulated kinase;
Glo-1:	glyoxalase 1;
HIF1:	hypoxia-inducible factor 1;
IL-6:	interleukin-6;
IRS-1:	insulin receptor substrate 1;
JNK:	c-Jun terminal kinases;
MAPK:	mitogen-activated protein kinase;
MCP-1:	monocyte chemoattractant protein-1;
MG:	methylglyoxal;
NFκB:	nuclear factor κB;
NRF2:	nuclear factor-erythroid 2-related factor-2;
PAS:	period acid-Schiff;
PKC:	protein kinase C;
RAGE:	receptor for advanced glycation endproducts;
ROS:	reactive oxygen species;
SAPK:	stress-activated protein kinases;
SREBP:	sterol regulatory element-binding protein;
T2D:	type 2 diabetes;
TLR4:	tool-like receptor 4;
TNFα:	tumor necrosis factor α;
WAT:	white adipose tissue
