# Supplementary material for: Adverse Effects of Methylglyoxal on Transcriptome and Metabolic Changes in Visceral Adipose Tissue in a Prediabetic Rat Model
Source: Antioxidants (Basel). 2020 Aug 31;9(9):803. doi: 10.3390/antiox9090803 (PMC7555565; doi:10.3390/antiox9090803)
Supplement: Supplementary file 1 [file antioxidants-09-00803-s001.pdf]

ID	Symbol	Entrez Gene Name	Expr p-value	Expr Fold Change
17835720	N.A.	N.A.	1.92E-07	1.40
17660843	NR1D1	nuclear receptor subfamily 1 group D member 1	6.06E-07	2.17
17847383	CISH	cytokine inducible SH2 containing protein	2.15E-06	-5.29
17818439	ENSRNOT00000081382	N.A.	2.69E-06	-1.18
17716660	ENSRNOT00000063100	N.A.	3.58E-06	1.16
17708216	LONRF1	LON peptidase N-terminal domain and ring finger 1	5.01E-06	-1,45
17691970	PUS10	pseudouridine synthase 10	5.20E-06	1,18
17821143	LPIN1	lipin 1	8.16E-06	1,31
17717621	SNORA17	Small nucleolar RNA SNORA17	8.73E-06	1,34
17690711	LRPAP1	LDL Receptor Related Protein Associated Protein 1	8.85E-06	-1,14
17838973	CACNG2	calcium voltage-gated channel auxiliary subunit gamma 2	1.01E-05	1,13
17684906	RGS2	regulator of G protein signaling 2	1.02E-05	1,66
17742419	BCAR3	BCAR3 adaptor protein, NSP family member	1,10E-05	1,27
17772052	ZEB2	zinc finger E-box binding homeobox 2	1.25E-05	-1,20
17645204	ADAM19	ADAM metallopeptidase domain 19	1.42E-05	-1,34
17814795	FAM49A	family with sequence similarity 49 member A	1.77E-05	1,40
17682903	DUSP10	dual specificity phosphatase 10	1.89E-05	1,63
17865965	IRS1	insulin receptor substrate 1	2.18E-05	-1,65
17877677	OTTRNOT00000000581	N.A.	2.23E-05	1,28
17659853	DYNLL2	dynein light chain LC8-type 2	2.39E-05	-1,16
17698672	C14orf119	chromosome 14 open reading frame 119	2.62E-05	1,28
17841570	ATF7	activating transcription factor 7	2.80E-05	1,12
17805044	DVL1	dishevelled segment polarity protein 1	2.93E-05	-1,18
17773291	ATF2	activating transcription factor 2	3.17E-05	1,10
17782888	Prss1 (includes others)	protease, serine 1 (trypsin 1)	3.41E-05	-1,33
17870740	LOC103690796	60S ribosomal protein L9-like	3.43E-05	1,25
17745872	RAI14	retinoic acid induced 14	4.15E-05	1,22
17715197	CAGE1	cancer antigen 1	4.37E-05	1,20
17721797	RNF125	ring finger protein 125	4.59E-05	1,33
17698930	GMPR2	guanosine monophosphate reductase 2	4.67E-05	1,07
17653649	RMI2	RecQ mediated genome instability 2	4.94E-05	-1,13
17649320	RHOT1	ras homolog family member T1	5.09E-05	1,16
17633469	SNORD115	Small nucleolar RNA SNORD115	5.10E-05	1,17
17810757	S100PBP	S100P binding protein	5.10E-05	-1,14
17783154	N.A.	N.A.	5.13E-05	-1,32
17662236	WNT9B	Wnt family member 9B	5.33E-05	-1,17
17779828	SYCP2	synaptonemal complex protein 2	5.53E-05	1,15
17628061	LPCAT1	lysophosphatidylcholine acyltransferase 1	5.64E-05	-1,24
17659429	TADA2A	transcriptional adaptor 2A	5.64E-05	1,23
17717253	Arl5b	ADP-ribosylation factor like GTPase 5B	5.69E-05	1,37
17698558	LOC290071	similar to T cell receptor V-alpha J-alpha	6.10E-05	1,30
17677021	TRIM50	tripartite motif containing 50	6.44E-05	-1,16
17691861	XPO1	exportin 1	6.71E-05	1,18
17645487	MGAT4B	alpha-1,3-mannosyl-glycoprotein 4-beta-N-acetylglucosaminyltransferase B	9.17E-05	-1,22
17860585	STK16	serine/threonine kinase 16	9.59E-05	1,14
17648510	mir-22	microRNA 22	9.65E-05	-1.31
17729005	GAB1	GRB2 associated binding protein 1	9.86E-05	-1,24
17671354	INSR	insulin receptor	1.02E-04	1,16
17872937	*Rpl34l1*	ribosomal protein L34-like1	1.03E-04	1,42
17635606	HBB	hemoglobin subunit beta	1.03E-04	1,31
17836388	OS9	OS9 endoplasmic reticulum lectin	1.05E-04	-1.10
17777243	CPXM1	carboxypeptidase X, M14 family member 1	1.15E-04	-1,63
17660371	ABI3	ABI family member 3	1.16E-04	1,31
17651031	KRTAP9-1	keratin associated protein 9-1	1.17E-04	1,25
17687416	IARS2	isoleucyl-tRNA synthetase 2, mitochondrial	1.18E-04	1,17
17794008	MBD4	methyl-CpG binding domain 4, DNA glycosylase	1.20E-04	1,17
17785859	ARL8B	ADP ribosylation factor like GTPase 8B	1.33E-04	1,15
17631580	LIN37	lin-37 DREAM MuvB core complex component	1.44E-04	1,11
17794371	WNK1	WNK lysine deficient protein kinase 1	1.50E-04	1,10
17849610	C11orf97	chromosome 11 open reading frame 97	1.51E-04	-1,22
17680884	TRMT1L	tRNA methyltransferase 1 like	1.57E-04	1,13
17640807	Olr327	olfactory receptor 327	1.63E-04	-1,17
17806228	N.A.	N.A.	1.66E-04	1,42
17707335	ZNF91	zinc finger protein 91	1.67E-04	1,28
17617874	PDE8A	phosphodiesterase 8A	1.68E-04	1,11
17870941	ENSRNOG00000053827	N.A.	1.82E-04	1,33
17877691	ENSRNOT00000078053	N.A.	1.99E-04	-1.20
17756992	DAXX	death domain associated protein	2.07E-04	1.13
17828543	LRIG3	leucine rich repeats and immunoglobulin like domains 3	2.13E-04	-1.26
17638636	IGF2	insulin-like growth factor 2	2.14E-04	-1.20
17612195	N.A.	N.A.	2.18E-04	-1,24
17804457	ENSRNOT00000078803	N.A.	2.20E-04	1,33
17877421	NDUFB4	NADH:ubiquinone oxidoreductase subunit B4	2.28E-04	1,45
17797247	TP53INP1	tumor protein p53 inducible nuclear protein 1	2.33E-04	1,34
17789572	PDK4	pyruvate dehydrogenase kinase 4	2.35E-04	1,76
17704951	TBC1D4	TBC1 domain family member 4	2.35E-04	-1.21
17864112	SNORD89	Small nucleolar RNA SNORD89	2.37E-04	-1.29
17789921	TSPAN12	tetraspanin 12	2.38E-04	1.17

Supplementary Table 1. List of transcripts significantly differentially expressed (FDR < 0.05) in white adipose tissue of adult HHTG male rats treated with methylglyoxal vs. control. ID refers to Affymetrix transcript ID, fold change indicates the change of expression in MG-treated vs. control rats. The analysis was performed using Partek Genomics Suite 7 (Partek Inc., St. Louis, MI, USA).
